# Qingjie Fuzheng Granule prevents colitis-associated colorectal cancer by inhibiting abnormal activation of NOD2/NF-*κ*B signaling pathway mediated by gut microbiota disorder

**DOI:** 10.1016/j.chmed.2025.04.001

**Published:** 2025-04-03

**Authors:** Bin Huang, Honglin An, Mengxuan Gui, Yiman Qiu, Wen Xu, Liming Chen, Qiang Li, Shaofeng Yao, Shihan Lin, Tatyana Aleksandrovna Khrustaleva, Ruiguo Wang, Jiumao Lin

**Affiliations:** a Academy of Integrative Medicine of Fujian University of Traditional Chinese Medicine, Fuzhou 350122, China; b Fujian Key Laboratory of Integrative Medicine on Geriatrics, Fujian University of Traditional Chinese Medicine, Fuzhou 350122, China; c The Second Affiliated Hospital of Fujian University of Traditional Chinese Medicine, Fuzhou 350122, China; d College of Pharmacy, Fujian University of Traditional Chinese Medicine, Fuzhou 350122, China; e Multidisciplinary Diagnostic Laboratory, Institute of Physiology of National Academy of Sciences of Belarus, Minsk 37517, Belarus

**Keywords:** colitis-associated colorectal cancer, gut microbiota, NOD2/NF-*κ*B pathway, Qingjie Fuzheng Granule, T cells

## Abstract

**Objective:**

This study investigates the efficacy and mechanisms of Qingjie Fuzheng Granules (QFG) in inhibiting colitis-associated colorectal cancer (CAC) development via RNA sequencing (RNA-seq) and 16S ribosomal RNA (rRNA) correlation analysis.

**Methods:**

CAC was induced in BALB/c mice using azoxymethane (AOM) and dextran sulfate sodium (DSS), and QFG was administered orally to the treatment group. The effects of QFG on CAC were evaluated using disease index, histology, and serum T-cell ratios. RNA-seq and 16S rRNA analysis assessed the transcriptome and microbiome change. Key pharmacodynamic pathways were identified by integrating these data and confirmed via Western blotting and immunofluorescence. The link between microbiota and CAC-related markers was explored using linear discriminant analysis effect size and Spearman correlation analysis.

**Results:**

Long-term treatment with QFG prevented AOM/DSS-induced CAC formation, reduced levels of interleukin (IL)-1*β*, tumor necrosis factor-alpha (TNF-*α*), IL-6, and interferon *γ* (IFN-*γ*), and increased CD3^+^ and CD4^+^/CD8^+^ T cells ratio, without causing hepatic or renal toxicity. A 16S rRNA analysis revealed that QFG rebalanced the Firmicutes/Bacteroidetes ratio and mitigated AOM/DSS-induced microbiota disturbances. Transcriptomics and Western blotting analysis identified the nucleotide-binding oligomerization domain-containing protein 2 (NOD2*)*/nuclear factor kappa-B (NF-*κ*B) pathway as key for QFG’s treatment against CAC. Furthermore, QFG decreased the abundance of Bacilli, Bacillales, Staphylococcaceae, *Staphylococcus*, Lactobacillales, *Aerococcus*, *Alloprevotella*, and *Akkermansia*, while increasing Clostridiales, Lachnospiraceae, Lachnospiraceae_NK4A136_group, Ruminococcaceae, and Muribaculaceae, which were highly correlated with CAC-related markers or NOD2/NF-*κ*B pathway.

**Conclusion:**

By mapping the relationships between CAC, immune responses, microbiota, and key pathways, this study clarifies the mechanism of QFG in inhibiting CAC, highlighting its potential for clinical use as preventive therapy.

## 1 Introduction

Colorectal cancer (CRC) is the third most prevalent cancer worldwide and the second leading cause of cancer deaths (Bray et al., 2024, Zheng et al., 2023). China ranks second in incidence and fourth in mortality, influenced by dietary changes (Han et al., 2024, Zheng et al., 2023). CRC development is associated with diet, age, inflammatory bowel disease (IBD), and intestinal dysbiosis (Cai et al., 2024, Davidson et al., 2021). Colitis-associated colorectal cancer (CAC) constitutes a significant subset of CRCs and a common complication of IBD (Sharma et al., 2018). Compared with the prevalent sporadic colorectal cancer, CAC progresses rapidly, has a higher malignant potential, lower survival rates, and poses significant therapeutic challenges (Plevris & Lees, 2022).

The primary therapeutic agents for IBD include 5-aminosalicylic acid, folic acid, ursodeoxycholic acid, and nonsteroidal anti-inflammatory drugs (Gupta et al., 2024, Wang et al., 2024). IBD requires long-term management due to its chronic course and tendency for recurrent flare-ups (Ashton et al., 2019). However, prolonged use of these medications can lead to cumulative toxic side effects, such as hepatotoxicity and neurotoxicity (Fardous & Heydari, 2023, Lin et al., 2019). Furthermore, there is a lack of clinical consensus on the potential of these medications to prevent IBD progression to CAC (Zhou et al., 2022). Therefore, strategies to prevent the onset and progression of CAC have significant implications for clinical interventions.

The interaction between microbiota pattern recognition receptors (PRRs) and the gut microbiome is crucial in promoting abnormal intestinal cell growth during recurring inflammation, facilitating the progression from IBD to CRC (Li et al., 2022). Nucleotide oligomerization domain (NOD)-like receptors (NLRs) are important PRRs that detect pathogenic microorganisms and transmit inflammatory signals. NOD1 can differentiate between peptidoglycan fragments derived from gram-negative and gram-positive bacteria. Conversely, NOD2 is considered a universal detector of gram-positive and gram-negative bacteria (de Zoete & Flavell, 2013). Nuclear factor kappa-B (NF-*κ*B) activation in intestinal epithelial cells (IECs) directly contributes to the onset of CAC by maintaining an inflammatory microenvironment via cytokine feedback regulation, which promotes excessive proliferation of IECs and intestinal mucosal cells, thereby facilitating the transition from IBD to CAC (Wei et al., 2023). The NOD1/2 activation triggers NF-*κ*B phosphorylation (Zhao et al., 2021).

Qingjie Fuzheng Granules (QFG) are derived from the “*Medical Records of the Qing Court*” and have been meticulously refined and optimized from the marketed LianQi Granules (B20020335). QFG prescription with *Hedyotis diffusa* Willd., *Scutellaria barbata* D. Don, *Astragali Radix* (Huangqi in Chinese) and *Hordei Fructus Germinatus* (Maiya in Chinese) primarily aims to clear heat, detoxify, and strengthen vital health and has extensive uses in cancer therapy (Huang et al., 2021). Previous studies indicated that QFG reduced intestinal damage, particularly when combined with 5-fluorouracil (5-FU), improving tumor suppression and minimizing 5-FU-related intestinal side effects (Zhang et al., 2019). Furthermore, QFG inhibits the migration and invasion of CRC cells. Clinically, QFG enhances colorectal cancer treatment, demonstrating notable efficacy in boosting therapeutic outcomes and reducing toxicity (Hua et al., 2019). However, the effect of QFG on CAC development remains unknown. This study aimed to clarify the role of QFG in inhibiting the progression of colitis to CRC and provide experimental evidence for its clinical use in CAC prevention.

## 2 Materials and methods

### 2.1 Preparation of QFG

QFG (Batch no. 2020002) is composed of *H. diffusa*, *S. barbata*, *Astragali Radix*, and *Hordei Fructus Germinatus* (1:1:1:1). The QFG was provided by the Pharmacy Unit of the People's Hospital affiliated to Fujian University, and its preparation process was as previously described (Lu et al., 2023). The UHPLC quality analysis was performed as follows: For the preparation of reference substances solutions, nine reference substances were separately weighed and dissolved in 70% methanol (UPLC-MS grade) to obtain single-stock solutions with precise concentrations. Each stock solution was diluted and mixed with 70% methanol to generate working standard solutions. Calibration curves were established using the mixed working standard solution, including hordenine 4 478 ng/mL, scutellarin 1 625 ng/mL, asperulosidic acid 2 425 ng/mL, asperuloside 551.80 ng/mL, ononin 3 260.8 ng/mL, astragaloside IV 284.0 ng/mL, baicalein 2 275 ng/mL, astragaloside I 1 241 ng/mL, and ursolic acid 207.6 ng/mL.

Quality sample solution of QFG: A total of 0.1 g of QFG powder was precisely weighed and transferred to a dark brown 50 mL calibrated flask. Subsequently, 40 mL of 70% methanol was added, and the mixture was sonicated for 30 min (power: 300 W, frequency: 40 kHz). The mixture was allowed to cool at room temperature. Subsequently, 70% methanol was added to bring the volume to the calibration mark on the flask. The extracted solution was centrifuged at 10 000 r/min for 10 min, and the supernatant was filtered using a 0.22 μm micropore membrane. Finally, the sample was diluted 10-fold by adding 900 μL of methanol solution to 100 μL of the sample solution.

### 2.2 Reagents and instruments

Azoxymethane (AOM, catalog no. A5486) and Ki-67 antibody (catalog no. AB9260) were obtained from Sigma Co., Ltd. (Livonia, USA). Dextran sulfate sodium (DSS, catalog no. 216011090) was sourced from MP Biomedicals (Santa Ana, USA). Occult blood reagent (Pilamitong semi-quantitative test, catalog no. BA-2020B) was bought from Zhuhai Beso Biotechnology Co., Ltd. (Zhuhai, China). Mouse interleukin (IL)-6 (IL-6) (catalog no. MM-0163M1), IL-1*β* (catalog no. MM-0040M1), tumor necrosis factor-alpha (TNF-*α*, catalog no. MM-0132ML), interferon *γ* (IFN-*γ*, catalog no. MM-0634ML), and enzyme-linked immunosorbent assay (ELISA) kit was acquired from Jiangsu Enzyme Immunity Industry Co., Ltd. (Yancheng, China). NOD1 (catalog no. sc-398696), NOD2 (catalog no. sc-56168) antibodies were received from Santa Cruz Biotechnology, Inc (Dallas, USA). NF-*κ*B p65 (mAb #8242), p-NF-*κ*B p65 (mAb #3033) antibodies were obtained from Cell Signaling Technology, Inc (Danvers, USA). TBP (22006-1-AP）polyclonal antibody was sourced from Proteintech Group, Inc (Wuhan, China). The reverse transcription reagents (RR047A) and SYBR Premix Ex Taq (RR420A) were procured from TaKaRa Co., Ltd. (Kusatsu, Japan).

### 2.3 UHPLC-MS analyses

An ACQUITY UHPLC I-Class system coupled with a Xevo XS quadrupole time of flight mass spectrometer (Waters, Milford, MA, USA) was used to characterize chemical components in QFG. Chromatographic separation was conducted at 45 °C on Waters CORTECS C_18_ column (100 mm × 2.1 mm, 1.6 μm). The mobile phases comprised 0.1% formic acid (phase A) and acetonitrile (phase B). Gradient elution was conducted as follows: 0–0.5 min, 5% B; 0.5–2 min, 5%–10% B; 2–4 min, 10%–40% B; 4–9 min, 40% B; 9–11 min, 40%–90% B; 11–13 min, 90%–90% B; 13–13.1 min, 90%–5% B; 13.1–15 min, 5% B. The flow rate was set to 0.2 mL/min, and the injected sample volume was 1 μL.

Mass spectrometry was performed using a Waters TQS triple quadrupole mass spectrometer (Waters, Milford, Massachusetts, USA) in positive ion mode with multiple reaction monitoring (MRM). The optimized MS conditions were fixed as follows: the capillary voltage was set at +3.00 kV; desolvation gas flow was 800 L/h (N2); desolvation gas temperature was 500 °C; source temperature was 150 °C; cone gas flow was 150 L/h (N2); collision gas was argon. Total MRM ion chromatograms are illustrated in Fig. S1. The calibration curves of detected compounds and calculated content of QFG are presented at in Table S1.

### 2.4 Animals and experimental design

Male BALB/c mice, 5–6 weeks old and weighing (22 ± 1) g, were obtained from Fujian University of Traditional Chinese Medicine's Laboratory Animal Center and housed in a controlled environment with a standard diet. The mice were subjected to a 1-week pre-feeding period to acclimate them to the experimental conditions. The animal experimental procedures were performed in accordance with the Guidelines for the Care and Use of Laboratory Animals formulated by the National Institutes of Health, and the protocols were approved by the Medical Ethics Committee of Fujian University of Traditional Chinese Medicine (Approval no. FJTCM IACUC 2020018).

**Fig. 1:**
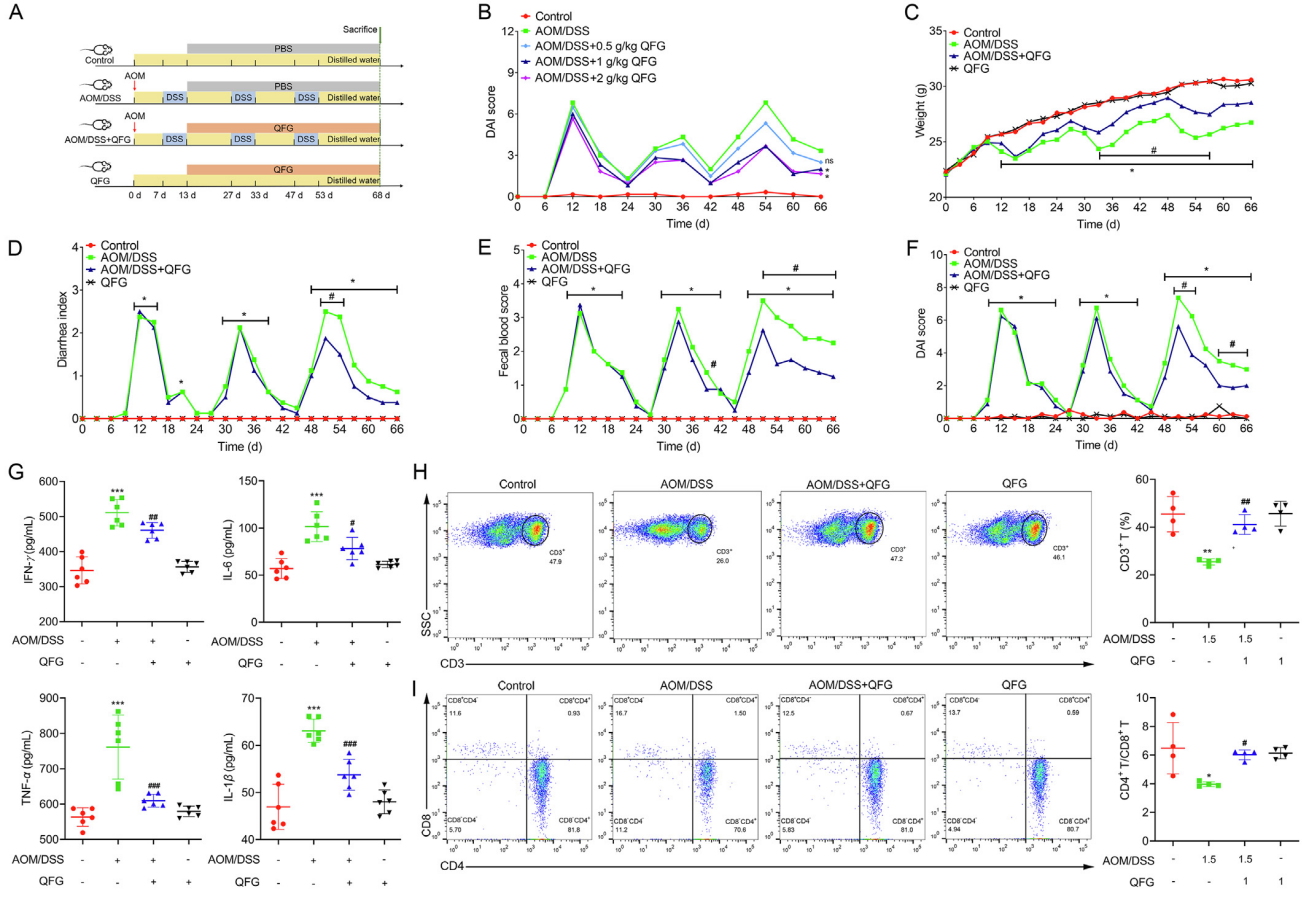
Effect of QFG on inflammatory symptoms and immune abnormalities in AOM/DSS-induced CAC mice. (A) Animal experimental protocol. (B) Effect of high, medium, and low doses of QFG on the DAI in mice. Body weight (C), average index of diarrhea (D), score for fecal blood (E), and DAI (F). Tukey's test and One-way ANOVA were employed to determine the statistical significance: *P < 0.05 *vs* control group; ^#^*P* < 0.05, ^##^*P* < 0.01 *vs* AOM/DSS group. (G) Creation of cytokines that cause inflammation (IFN-*γ*, IL-6, TNF-*α*, and IL-1*β*) in mice's serum (*n* = 6). ^***^P < 0.001 *vs* control group, ^#^*P* < 0.05, ^##^*P* < 0.01 *vs* AOM/DSS group. (H − I) Proportion of CD3^+^, CD4^+^, and CD8^+^ T cell subsets in mouse blood (*n* = 4). Games–Howell test or Tukey's test and One-way ANOVA were used to determine the statistical significance: *P < 0.05, ^**^P < 0.01 *vs* control group, ^#^*P* < 0.05, ^##^*P* < 0.01 *vs* AOM/DSS group.

A total of 32 mice were divided into four groups (with eight mice in each group) based on body weight: Control, AOM/DSS, AOM/DSS + QFG, and QFG. The control group received regular water. AOM/DSS and AOM/DSS + QFG groups were administered AOM (12.5 mg/kg) injection and, after 7 d, drank 2.5% DSS water for 6 d, followed by regular water for 14 d; this process was repeated three times. Following the first DSS cycle, the AOM/DSS + QFG group received QFG (1 g/kg). The QFG group drank only water and received oral QFG (1 g/kg). At the end of the experiment, after 16 h of fasting, all mice were euthanized, colon segments were preserved in 4% paraformaldehyde, and tissues were stored at –80 °C for subsequent RNA sequencing (RNA-seq) or Western blotting analysis. This procedure is illustrated in Fig. 1A.

### 2.5 Observation of weight loss, diarrhea, and fecal blood score in each group

Daily monitoring included the weight and fecal quality of each mouse. A standardized blind assessment protocol (Zhong et al., 2024) was used to assess diarrhea severity, fecal occult blood (FOB), and disease activity index (DAI). The DAI composite score includes FOB, diarrhea severity, and weight loss.

### 2.6 Histological examination

The hematoxylin (G1140) and eosin (G1100) (H&E) stains used in this study were obtained from Beijing Solarbio Science and Technology Co., Ltd. (Beijing, China). The colon tissue samples from each mouse were embedded in paraffin, sliced into 4 µm sections, and stained with H&E for microscopic evaluation of villus height, crypt depth, and crypt orientation.

### 2.7 Preparation of colonic tissue nuclear protein samples

A total of 30–80 mg of colonic tissue was taken from the –80 °C freezer and transferred into a 2 mL centrifuge tube with grinding beads. A total of 600 μL of pre-cooled hypotonic buffer was added to the tissues. The mixture was homogenized using a pre-cooled tissue grinder three times (70 Hz, 1 min each). Afterward, the mixture was centrifuged (3 000 r/min for 5 min at 4 °C, and the supernatant was discarded. A total of 400 μL of hypotonic buffer was added, vortexed for 30 s, centrifuged again at 5 000 r/min for 5 min at 4 °C, discarded the supernatant, and retained the pellet. Subsequently, 0.2 mL of lysis buffer was added to the pellet, vortexed for 30 s, incubated on ice for 20 min, and centrifuged (15 000 r/min for 20 min at 4 °C). The supernatant was collected and transferred to a 1.5 mL centrifuge tube for further experiments or stored at –80 °C.

### 2.8 Western blotting

Total protein was extracted from colon samples and quantified using the BCA assay. Protein lysates were separated using SDS-PAGE, transferred to a nitrocellulose membrane, and probed with primary antibodies against NOD1, NOD2, NF-*κ*B p65, p-NF-*κ*B p65 (Ser536), TBP, and GAPDH at dilutions of 1:1 000, 1:1 000, 1:2 000, 1:1 000, 1:3 000, and 1:10 000, respectively. The membranes were subsequently incubated with secondary antibodies (dilution: 1:10 000; Thermo Fisher Scientific, Waltham, MA, USA) and chemiluminescence was detected. Protein band intensity was analyzed using Image Lab software (Bio-Rad, Hercules, CA, USA).

### 2.9 Immunohistochemistry (IHC)

Immunohistochemical staining was performed using a kit (Kit-0017) from Maixin Biotechnology (Fuzhou, China), with colon tissues sectioned and prepared for staining. The sections were treated with microwave heating in citrate buffer and incubated with the Ki-67 antibody (1:200). Secondary antibodies were applied, followed by hematoxylin counterstaining and DAB staining. The sections were subsequently dehydrated, sealed, and analyzed under a microscope using Image-Pro Plus software (version 5.1).

### 2.10 ELISA

The ELISA kits for IL-6, IL-1*β*, TNF-*α*, and IFN-*γ* were sourced from Jiangsu Meimian Industrial Co., Ltd. (Nanjing, China). Blood was collected from euthanized mice, which were allowed to clot and centrifuge to separate the serum. Cytokine levels were quantified according to the manufacturer's protocol, with optical density values measured spectrophotometrically using a standard curve.

### 2.11 T lymphocyte subsets assay

The T lymphocyte subsets, including CD3^+^, CD4^+^, and CD8^+^, were analyzed from blood collected from the mice in ethylenediaminetetraacetic acid-containing tubes using a BD FACSCelesta flow cytometer (BD Biosciences, USA).

### 2.12 16S rRNA gene sequencing of mice feces

16S rRNA gene sequencing and statistical analysis were conducted with reference to previously described (Huang et al., 2022). The mouse fecal samples were stored at –80 °C, transferred to a mortar, and ground into a fine powder. DNA was extracted using a fecal DNA kit (MN, Germany) following the provided protocol. The V3-V4 region of the 16S rRNA gene was amplified using the 341F/806R primer set, as previously described (Matsuo et al., 2021). Amplified DNA was purified and quantified using a NanoDrop 2000 spectrophotometer. Finally, the purified polymerase chain reaction products were sequenced using high-throughput technology (BiomMarker Technologies Corporation) on an Illumina HiSeq2500 platform.

### 2.13 RNA-seq and enrichment analysis

RNA-seq and enrichment analysis were conducted reference to previously described (Romero et al., 2022). The samples were sequenced on an Illumina platform to assess gene expression levels across the groups. The “limma” package was used for analyses involving biological replicates, and “DESeq” was employed for comparison of non-biological replicates. DESeq has been consistently employed in mixed projects. Differentially expressed genes (DEGs) were identified based on the following criteria: |log_2_FC| ≥ 1.2, *P* < 0.05.

Enrichment analysis was performed using a hypothesis testing approach based on a hypergeometric distribution to determine the *P*-value of the enrichment results. The *P*-value was adjusted using the Benjamini-Hochberg method for multiple hypothesis testing to minimize false positives. A corrected *P*-value of < 0.05 was considered statistically significant, with smaller values indicating more significant enrichment.

### 2.14 Immunofluorescence (IF) staining

Tissue sections were blocked with 5% goat serum and 0.015% TritonX-100 for 1 h at room temperature, followed by overnight incubation at 4 °C with the NF-*κ*B p65 primary antibody (mAb#8242, 1:200, CST, USA). Following three phosphate buffered saline washes (5 min each), the sections were incubated with an FITC-conjugated secondary antibody, Affinipure goat anti-rabbit IgG (H + L) (SA00003-2, 1:500 dilution, Proteintech, China), for 1 h at room temperature. Ultimately, the sections were stained with DAPI (2-(4-Amidinophenyl)-6-indolecarbamidine dihydrochloride) and imaged using a laser scanning confocal microscope (LSM710; Zeiss, Germany).

### 2.15 Statistical analyses

The data are presented as the mean ± standard deviation. Histograms were generated using GraphPad Prism software (version 9.4.2; La Jolla, CA, USA). All statistical analyses were performed using Statistical Package for the Social Sciences software (version 23.0; SPSS, Inc., Chicago, IL, USA). Multiple group comparisons were analyzed via One-way ANOVA, with post hoc comparisons conducted using either Tukey's test (assuming equal variances) or the Games-Howell test (for heterogeneous variances), as applicable. Comparisons of the two groups were performed using a two-tailed unpaired *t*-test. Spearman correlation coefficient was used to assess parameter relationships. Results were considered statistically significant at *P* < 0.05.

## 3 Results

### 3.1 QFG treatment alleviated recurrent colitis symptoms during AOM/DSS-induced CAC development

A CAC mouse model was established (Fig. 1A). The optimal concentration of 1 g/kg for QFG treatment was selected by a preliminary experiment consisting of high, medium and low doses of QFG before the experiment began (Fig. 1B). Following six days of DSS administration, mice exhibited IBD-like symptoms, including weight loss, diarrhea, and blood in the feces, mimicking the initial flare-up of IBD in clinical situations. QFG treatment in the AOM/DSS + QFG group significantly reduced the modeling agent's suppressive effects on weight gain, with notable improvement observed by day 36 (*P* < 0.05). From day 9, the AOM/DSS group exhibited worse diarrhea and fecal blood scores compared to the control (*P* < 0.05); however, long-term QFG therapy alleviated these symptoms during the third DSS challenge (*P* < 0.05). Overall, QFG treatment reduced DAI during the third exposure to the CAC modeling agent (*P* < 0.05). Mice receiving only QFG demonstrated no adverse symptoms (Fig. 1C–F).

### 3.2 Effects of QFG treatment on pro-inflammatory cytokine release in CAC mice serum

AOM/DSS modeling significantly increased TNF-*α*, IFN-*γ*, IL-1*β*, and IL-6 levels compared to the control group (*P* < 0.05). QFG treatment significantly reduced the levels of these pro-inflammatory cytokines compared to the AOM/DSS group (*P* < 0.05). Long-term QFG treatment alone maintained cytokine levels similar to those in the control group (*P* > 0.05) (Fig. 1G).

### 3.3 Effects of QFG on T lymphocyte population against AOM/DSS treatment

Studies have demonstrated alterations in T cell subsets in patients with CAC (Olguin et al., 2018, Zhang et al., 2018). The findings of this study revealed that AOM/DSS treatment significantly reduced CD3^+^ T cell growth and the CD4^+^/CD8^+^ subset ratio compared to controls (*P* < 0.05). Furthermore, QFG treatment effectively reversed these T cell disruptions compared to the AOM/DSS group (*P* < 0.05). Long-term QFG treatment alone maintained T cell subsets comparable to those in the controls (*P* > 0.05) (Fig. 1H–I).

### 3.4 Long-term QFG treatment ultimately inhibited formation of CAC

**Fig. 2:**
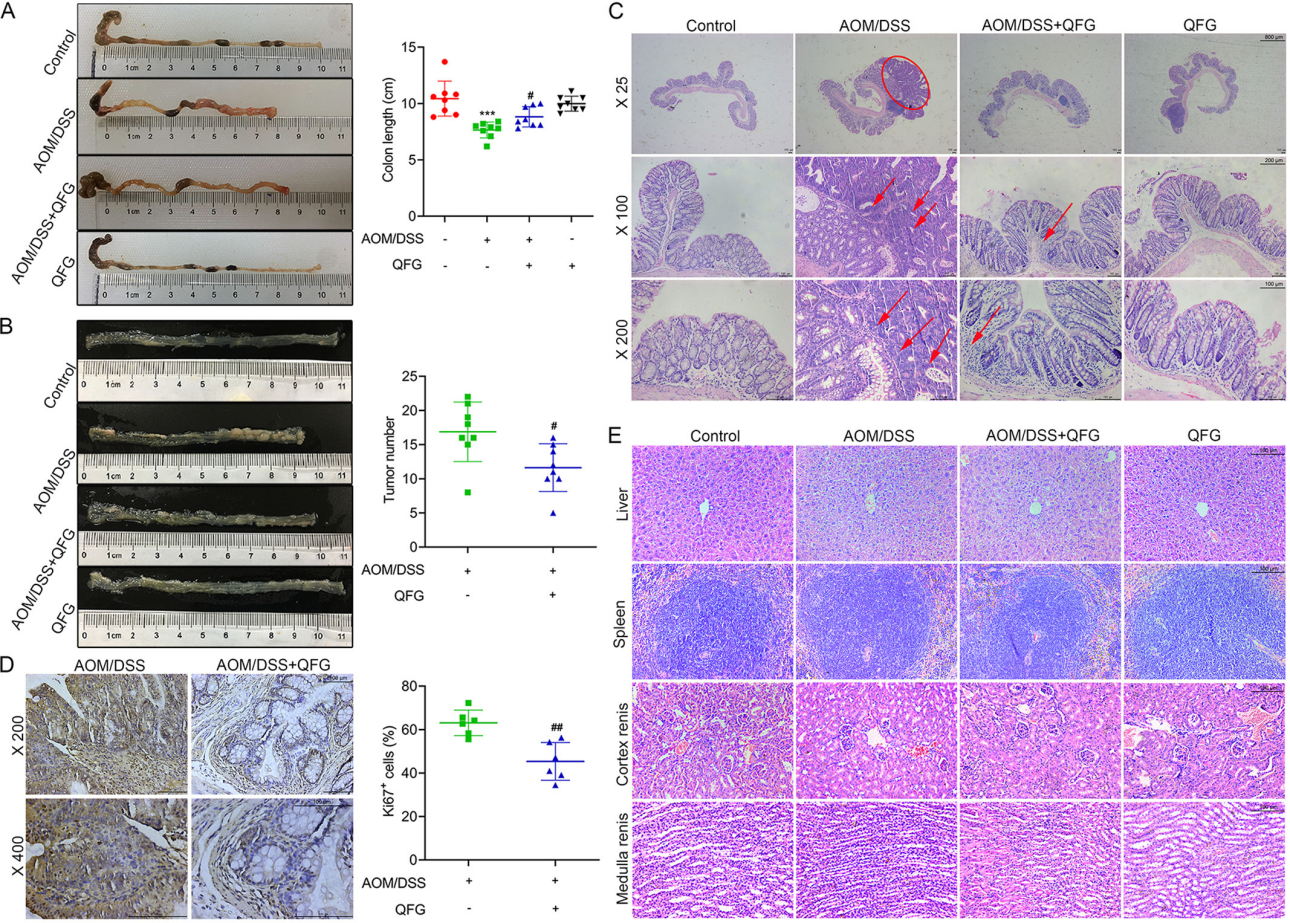
Inhibitory effect of long-term QFG administration on CAC tumor formation and its toxic side effects. (A) Colon lengths of each group were measured and contrasted using graphs of the average colon length and an image (*n* = 8). One-way ANOVA and Tukey's test were used to determine the statistical significance: ^***^P < 0.001 *vs* control group, ^#^*P* < 0.05 *vs* AOM/DSS group. (B) Tumor numbers of each group were measured and contrasted using graphs of the colon anatomy (*n* = 8). Student's *t*-test was utilized to determine the statistical significance: ^#^*P* < 0.05 *vs* AOM/DSS group. (C) Representative histological slices of the colon from each group were stained with HE (× 25, scale bar = 800 μm; × 100, scale bar = 200 μm; × 200, scale bar = 100 μm). (D) Expression of Ki67 in representative colonic tissues detected by IHC. (× 200, scale bar = 100 μm; × 400, scale bar = 100 μm). Student's *t*-test was utilized to determine the statistical significance: ^##^*P* < 0.01 *vs* AOM/DSS group. (E) Sections of the liver, spleen, and kidney in each group were stained with HE (× 200, scale bar = 100 μm).

The AOM/DSS model induced colonic inflammatory injury and CAC. Treatment with QFG prevented colon shortening and reduced tumor development compared to the AOM/DSS group (*P* < 0.05) (Fig. 2A and B). Histological analysis revealed normal colon structures in the control group, while CAC mice exhibited severe inflammation and tumors characterized by crypt defects, goblet cell depletion, disordered epithelial arrangements, significant inflammation, excessive hyperplasia, and colon carcinoma development (Fig. 2C). QFG treatment significantly improved colon histopathology by reducing tumor size and number and inhibiting tumor cell proliferation (*P* < 0.05) (Fig. 2C and D).

### 3.5 Evaluation of toxic side effects with long-term QFG administration

Compared with the control group, the spleen weight and spleen index of the AOM/DSS model group were significantly increased, and both were significantly restored after QFG treatment (*P* < 0.05). At the same time, the liver weight of the model group was significantly reduced (*P* < 0.05). After QFG intervention, and there was no significant difference between the model group and the control group (*P* > 0.05). In addition, there were no differences in liver index, kidney weight, and kidney index among the control, model, and QFG treatment groups (*P* > 0.05) (Fig. S2), further histopathology revealed no significant changes in the histomorphology of liver, spleen and kidney in either group (Fig. 2E). This experiment required over 50 d of continuous QFG administration to inhibit the progression from IBD to CAC. Consequently, a QFG group was established to assess the adverse impact of long-term QFG use. Histological findings indicated that long-term QFG in non-model conditions did not adversely affect the liver, spleen, or kidneys compared to the controls (Fig. 2E).

### 3.6 QFG reduced gut microbiota dysbiosis induced by AOM/DSS

**Fig. 3:**
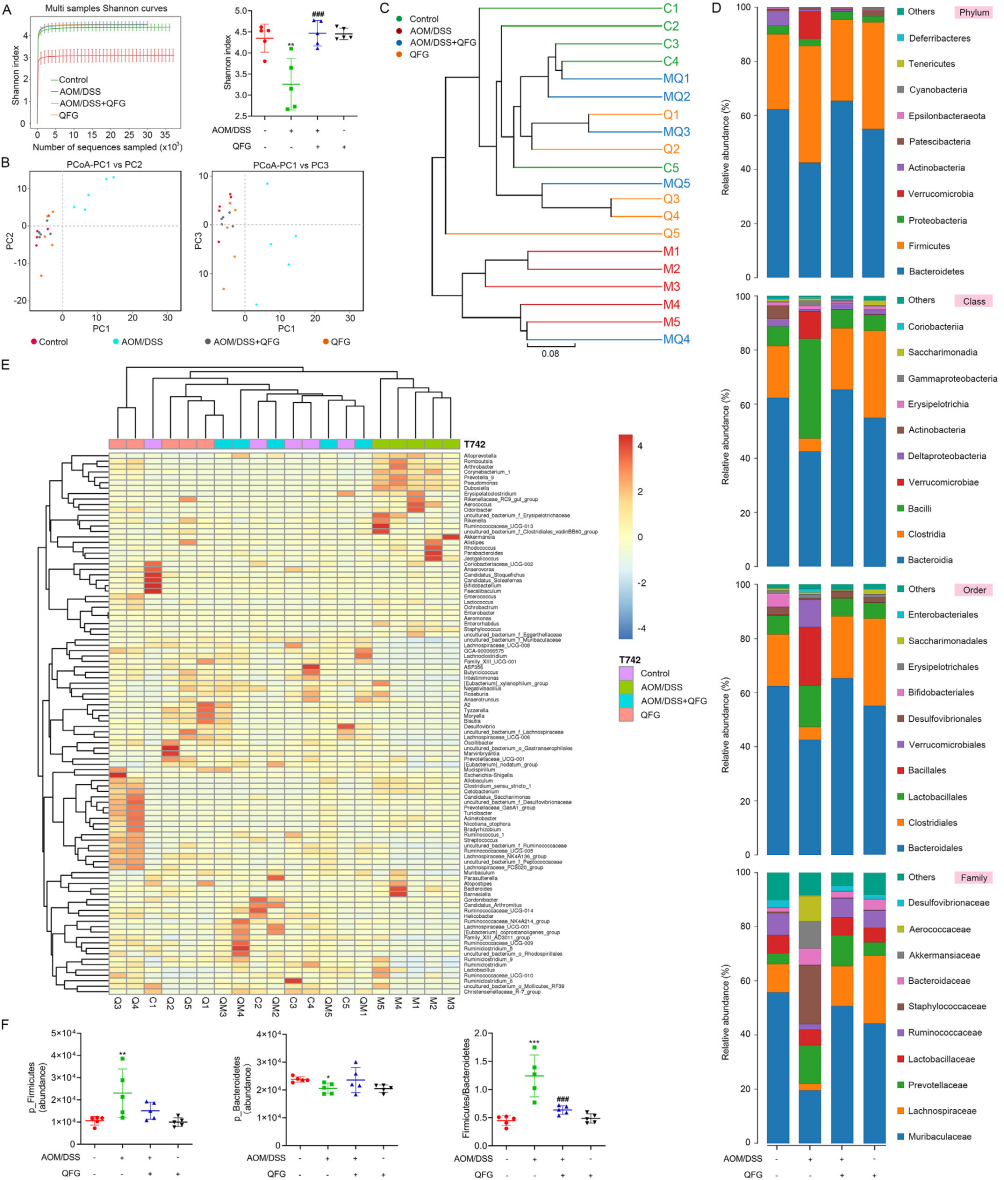
Effects of QFG on microbial disorder and relative abundance of top ten most abundant microbiota taxa in CAC mice. (A) Alpha diversity was calculated according to the Shannon diversity index. (B) PCoA plots were used to visualize beta diversity. (C) Clustering diagram of microbiota communities in mice. (D) Abundance histogram of intestinal microflora in mice at phylum, class, order, and family level. (E) A histogram of the abundance of intestinal microflora in mice at genus level. (F) Statistics of Firmicutes and Bacteroidetes in mice. One-way ANOVA and Tukey's test were used to determine the statistical significance: *P < 0.05 ^**^P < 0.01 ^***^P < 0.001 *vs* control group, ^###^*P* < 0.001 *vs* AOM/DSS group.

Food and water intake were standardized to minimize variations in energy intake and its effects on gut microbiota. The Shannon index, a measure of species diversity, was utilized. A significant reduction in the Shannon curve and index for the gut microbiota was observed in the AOM/DSS group compared to the control group (*P* < 0.05) (Fig. 3A). Long-term QFG treatment significantly increased intestinal microbiota abundance in the AOM/DSS + QFG group (*P* < 0.05). Principal coordinate analysis (PCoA) of the Bray-Curtis tree evaluated bacterial community diversity and highlighted species similarities among samples. The AOM/DSS group demonstrated notable deviations, whereas the microbial patterns of the AOM/DSS + QFG group closely resembled those of the controls. Long-term QFG consumption induced no significant changes in intestinal flora compared to the controls (Fig. 3B and C).

The abundance of the top 10 taxa was evaluated to identify gut microbiota changes (Fig. 3D). In phylum-level analysis, the AOM/DSS group revealed a significant increase in Firmicutes compared to the controls (*P* < 0.01) (Fig. 3F). The Firmicutes to Bacteroidetes (F/B) ratio is commonly utilized to assess the effect of gut microbiota on various diseases (Bahar-Tokman et al., 2022). Notably, the F/B ratio in AOM/DSS group was significantly higher than that in the control group (Fig. 3F). In class-level analysis, the AOM/DSS group showed a decrease in Bacteroidia and Clostridia compared to the controls, and an increase in Verrucomicrobiae and Bacilli. In the order-level analysis, the AOM/DSS group exhibited decreased numbers of Bacteroidales and Clostridiales and increased numbers of Verrucomicrobiales, Bacillales, and Lactobacillales compared to the controls. In the family-level analysis, the AOM/DSS group revealed reduced numbers of Muribaculaceae, Lachnospiraceae, and Ruminococcaceae while increased numbers of Aerococcaceae, Akkermansiaceae, and Staphylococcaceae compared with the controls. In the genus-level analysis, the AOM/DSS group exhibited decreased levels of Lachnospiraceae_NK4A136_group and increased *Aerococcus*, *Akkermansia*, *Staphylococcus*, and *Alloprevotella* compared to the controls (Fig. 3E). These changes were reversed in the AOM/DSS + QFG compared to the AOM/DSS group. Furthermore, no significant alterations in intestinal microbiota were observed in mice that received long-term QFG without modeling compared to the controls.

### 3.7 QFG suppresses NOD2/NF-κB signaling activation, aligning with transcriptomic results

**Fig. 4:**
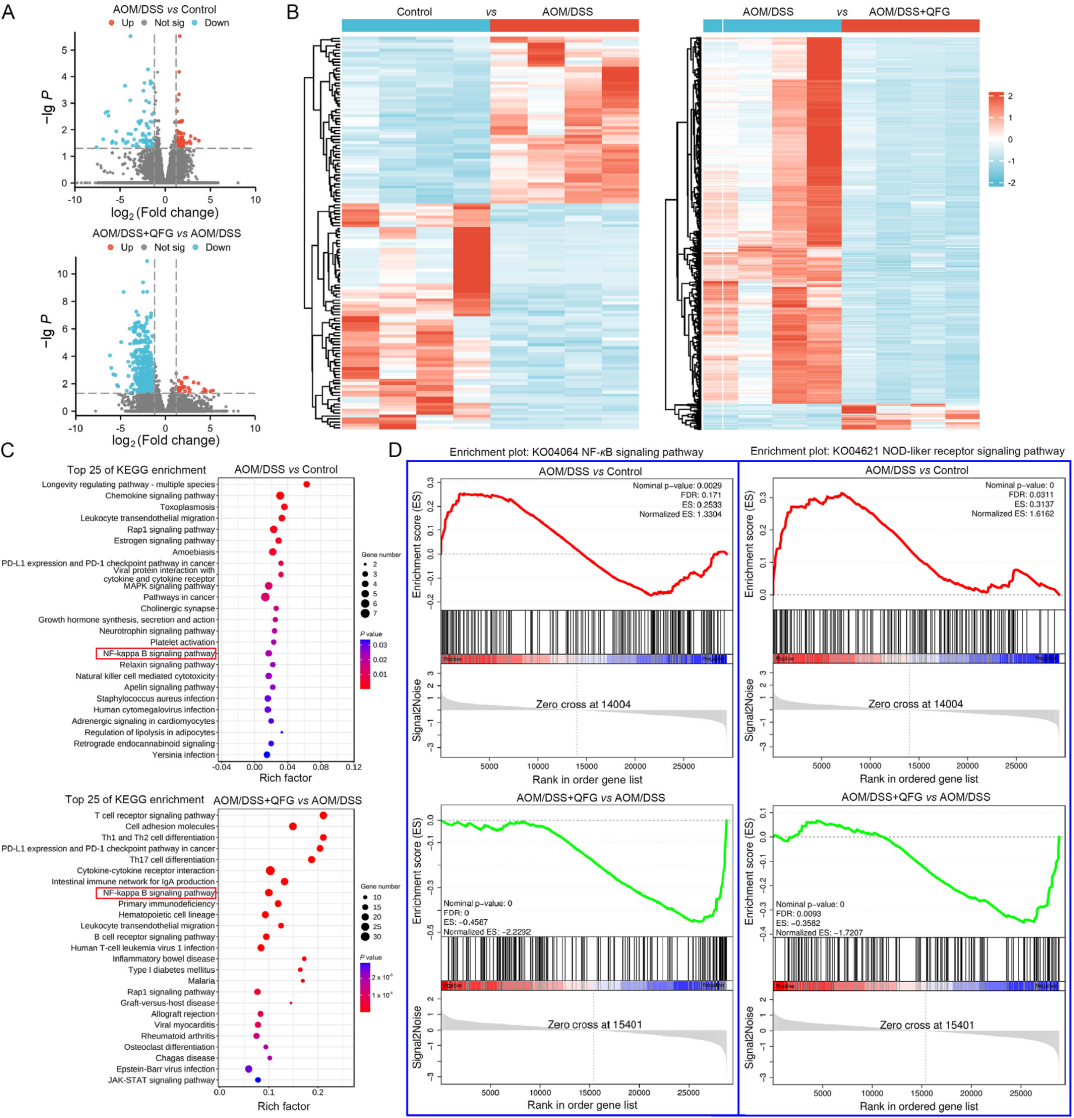
Key pharmacological mechanism of QFG confirmed by transcriptomic study. (A) Volcano plot of differential genes. (B) Cluster heatmaps of DEGs. (C) Pathway enrichment analysis performed to identify the related KEGG pathways. (D) Gene set enrichment analysis of NF-*κ*B and NOD-like receptor signaling pathways.

The primary targeted pathway through which QFG inhibits the progression from IBD to CAC was analyzed using RNA-seq and bioinformatics mining. This involved comparisons between the control and the AOM/DSS groups and between AOM/DSS + QFG and AOM/DSS groups (Fig. 4A and B). Notably, the “NF-*κ*B signaling pathway” played a crucial role in mediating the pharmacological effects of QFG in preventing the progression of IBD to CAC (Fig. 4C).

Understanding the relationship between recurrent IBD and CAC is crucial. In this context, the continuous activation of NF-*κ*B (p65) along with increased levels of pro-inflammatory cytokines such as TNF-*α*, IL-1*β*, INF-*γ*, and IL-6 are essential (Cuellar-Nunez et al., 2021, Lu et al., 2024). These factors contribute to a chronic inflammatory environment that accelerates CAC onset. NF-*κ*B influences T cell behavior, promoting immune evasion and tumor growth (Xiang et al., 2024). Inhibiting the “NF-*κ*B signaling pathway” can enhance T cell efficacy and prevent CAC (Wang et al., 2019). Our findings indicated that QFG can inhibit pro-inflammatory cytokines and improve T-cell imbalance caused by AOM/DSS. RNA-seq results supported that QFG's inhibition of CAC progression primarily occurs via the NF-*κ*B signaling pathway.

**Fig. 5:**
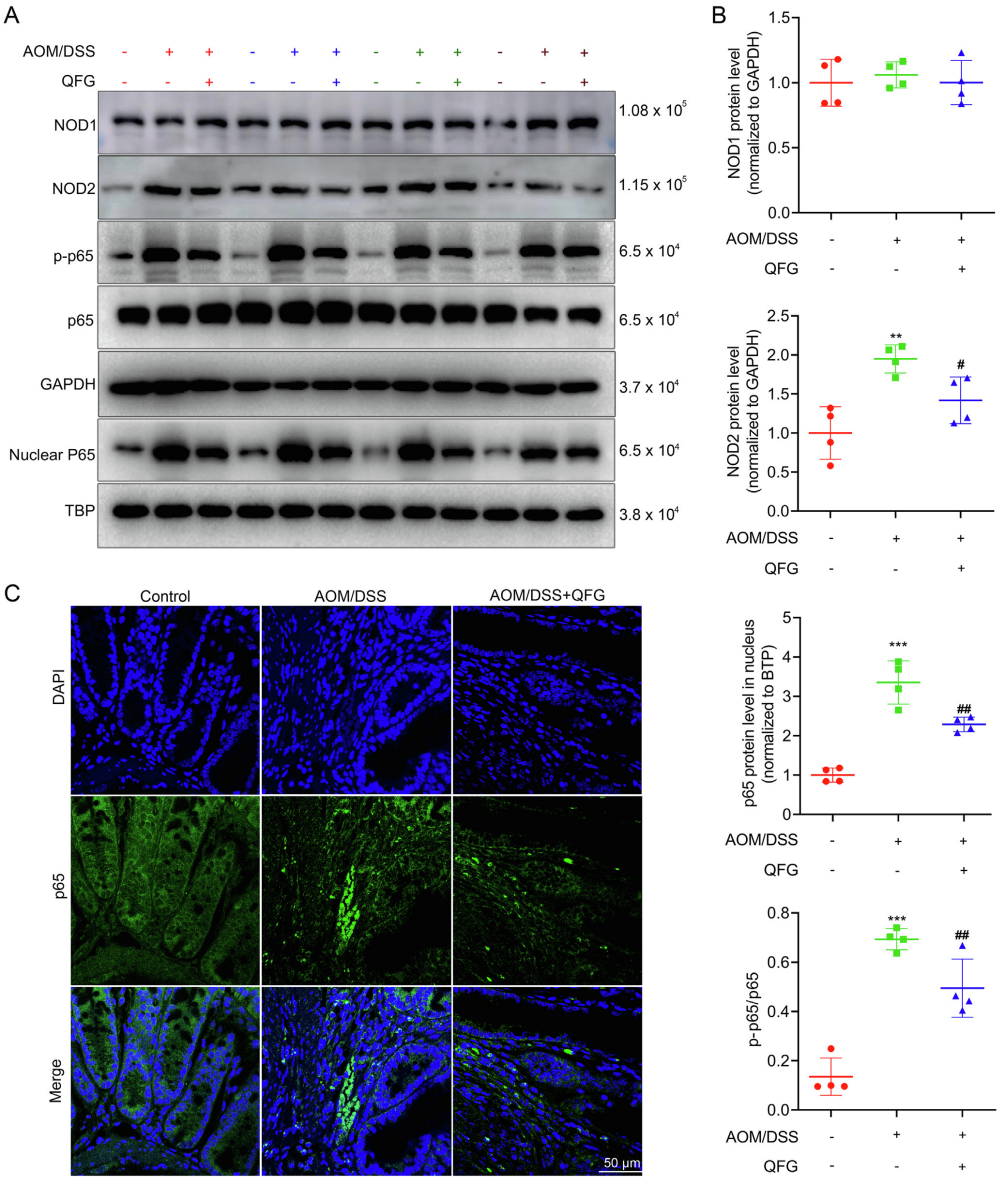
QFG inhibited NOD2/NF-*κ*B signaling pathway. Western blotting images (A) and gray-scale statistics (B) of signaling pathway related proteins in mouse colon tissue. (C) Distribution of FITC-labeled p-NF-*κ*B p65 in the colon of mice (× 400, scale bar = 50 μm). One-way ANOVA and Tukey's test were used to determine the statistical significance: ^**^P < 0.01 ^***^P < 0.001 *vs* control group, ^#^*P* < 0.05 ^##^*P* < 0.01 *vs* AOM/DSS group.

Considering QFG's positive impact on gut microbiota, RNA-seq enrichment of pattern recognition receptors (PRR)-associated pathways was analyzed, revealing a significant regulation in the “NOD-like receptor signaling pathway” by QFG. This suggested NODs/NF-*κ*B pathway involvement in QFG's anti-CAC mechanism (Fig. 4D). WB or IF evaluated the expressions of NOD1, NOD2, and p-NF-*κ*B p65/NF-*κ*B p65 in mouse colon tissues (Fig. 5A–C). CAC mice demonstrated increased NF-*κ*B phosphorylation and NOD2 expression, with a significant rise in nuclear NF-*κ*B p65 (*P* < 0.05) compared to the control group. NOD1 expression remained unchanged (*P* > 0.05). QFG treatment at 1 g/kg downregulated NOD2 and decreased NF-*κ*B p65 activation compared to the AOM/DSS group (*P* < 0.05).

### 3.8 Investigating interaction between biological parameters within gut microbiota

**Fig. 6:**
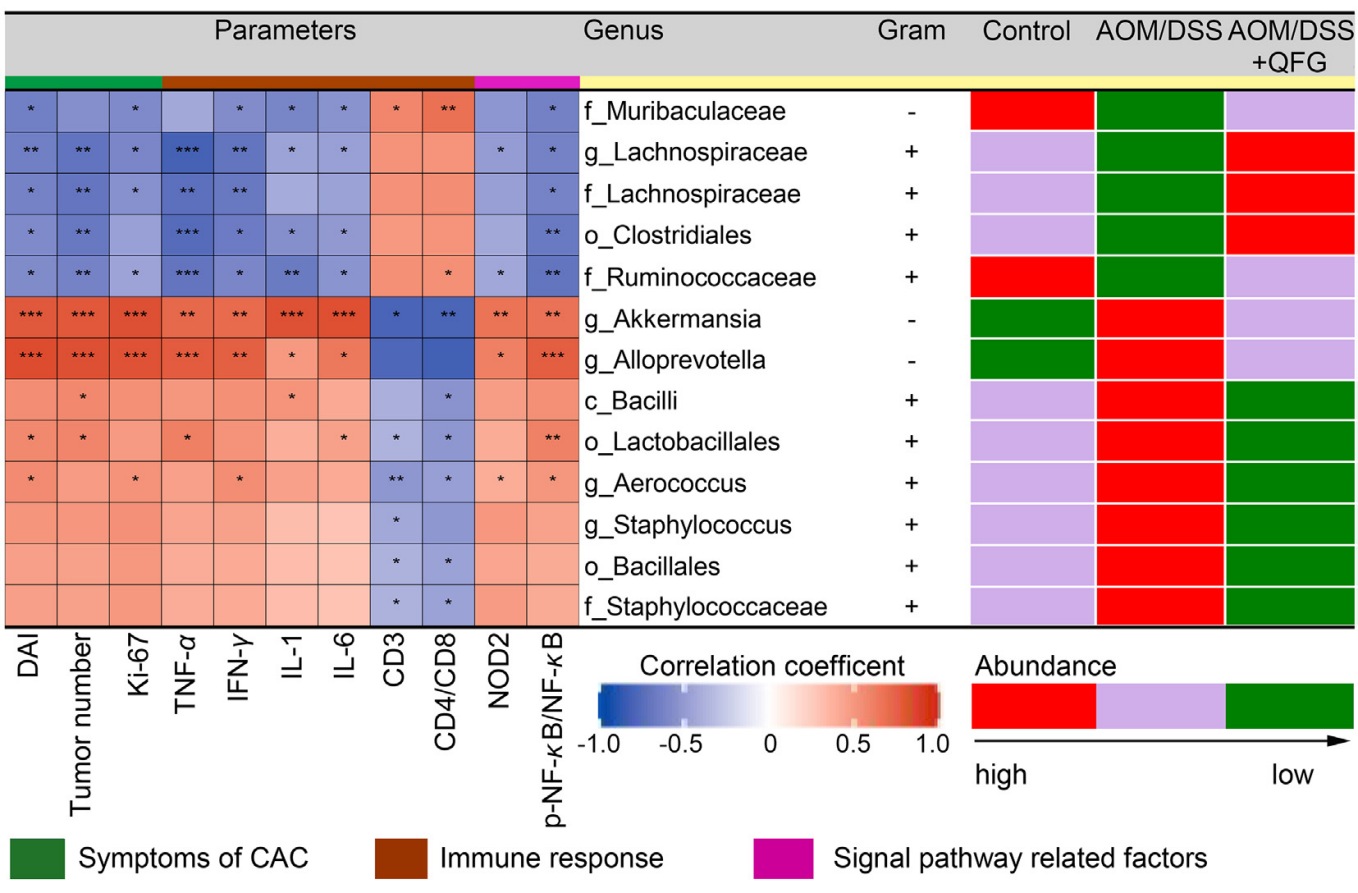
Investigation of relationships between biological markers and gut microbiota. The correlation coefficients between gut microbiota and biological markers are depicted on a heatmap. *R*^2^ > 0.25 denotes moderate correlation, while *R*^2^ > 0.36 indicates a significant connection in the correlation coefficient. Significant correlations are indicated by asterisks (*P < 0.05, ^**^P < 0.01, ^***^P < 0.001). The positive and negative correlations are presented in red and blue, respectively. Stronger relationships are denoted by darker hues. The relative abundance of the relevant bacteria was high, medium, and low in the control, AOM/DSS, and AOM/DSS + QFG groups, respectively, as denoted by the red, purple, and green squares. (For interpretation of the references to colour in this figure legend, the reader is referred to the web version of this article.)

The Spearman correlation coefficient, linear discriminant analysis (LDA) effect size (LEfSe) methods were used to construct a correlation matrix at the taxonomic levels of class, order, family, and genus to investigate the relationships between the 13 bacteria and nine distinct parameters (Fig. 6). Each of the 13 bacteria demonstrated positive or negative correlations with at least one AOM/DSS-induced CAC parameter, colitis/CRC indicators, or genes related to the NOD2/NF-*κ*B signaling pathway. In this context, the abundances of eight bacteria were increased in the gut microbiota of CAC mice, while the abundances of the remaining five bacteria were decreased. However, following long-term intervention with QFG, the relative abundance of these bacteria was notably reversed in the AOM/DSS + QFG group compared to the AOM/DSS group. These findings highlighted the critical roles of inflammation, immune response, gut microbiota, and the NOD2/NF-*κ*B pathway in inhibiting CAC development by QFG.

## 4 Discussion

CRC is the third most common cancer worldwide and the second leading cause of cancer-related deaths (Bray et al., 2024). China has the highest CRC rates globally, especially with a rising incidence among men, despite a global decline (Han et al., 2024). Chronic inflammation, particularly in patients with IBD, significantly increases CRC risk, with 13.9% of Asian patients with IBD developing CRC after 30 years (Shah & Itzkowitz, 2022). Chronic colonic inflammation can lead to CAC, which often progresses into aggressive signet ring cell carcinoma and affects younger individuals (Shah & Itzkowitz, 2022). Managing inflammation and IBD symptoms such as diarrhea, fecal blood loss, and weight loss is essential for preventing CAC (Jin et al., 2024, Zhao et al., 2024). However, common IBD treatments, including 5-ASA, methotrexate, azathioprine, and 6-mercaptopurine, have not demonstrated efficacy in preventing CAC, and their prolonged use may cause organ damage (Yan et al., 2024, Zhou et al., 2022).

Integrative oncology, which incorporates traditional Chinese medicine (TCM), is widely utilized in cancer treatment (Cai et al., 2024, Fu et al., 2022). The TCM principle of “prevention before illness and prevention of progression once ill” is applied in tumor prevention: TCM is used to prevent cancer before its onset, and a combination of traditional Chinese and Western medicine is used for treatment after diagnosis (Kong et al., 2023, Liu et al., 2024). Our team has developed an innovative TCM called QFG, used alongside chemotherapy for colon cancer treatment in several hospitals in Fujian Province (Hua et al., 2019). QFG inhibits CRC, increases chemotherapeutic efficacy, decreases intestinal inflammation (Zhang et al., 2019), and improves postoperative immune function (Zhong et al., 2024). However, further research is needed to assess the potential of QFG in preventing CAC development or colitis progression to colon cancer.

In TCM, the interplay between phlegm and blood stasis, coupled with toxic pathogen accumulation, is considered fundamental to the onset of CAC (Sun et al., 2016). TCM emphasizes the preventive and therapeutic approaches for CAC, including clearing heat, removing toxins, softening hardness, dissipating masses, eliminating pathogenic factors, and reinforcing healthy *qi* (Yue et al., 2024, Huang et al., 2023). Among the components of QFG, *H. diffusa* and *S. barbata* are used to clear heat and detoxify, as well as to soften hardness and disperse masses, while *Astragali Radix* and *Hordei Fructus Germinatus* can strengthen the spleen and replenish healthy *qi*, aligning with these principles. and we employed an AOM/DSS mouse model to study the progression of colitis into cancer. We simulated the real-world scenario of initiating treatment following the first clinical onset of IBD. The QFG intervention (via gastric gavage) was administered at the end of the first DSS cycle, followed by long-term medication during recurrent IBD episodes to evaluate its efficacy in preventing colonic “inflammation-cancer transformation.” QFG decreased AOM/DSS-induced symptoms, including bloody diarrhea and weight loss in mice. It also reduces colon damage and tumor growth. Overall, QFG effectively prevented CAC in recurrent IBD cases.

Patients with IBD require prolonged treatment to prevent colitis from developing into CAC (Ashton et al., 2019). A long-term QFG group was established in mice to assess the adverse effects of prolonged QFG intake. Additionally, we observed how the liver, kidneys, and spleen were affected in mice treated with AOM/DSS + QFG compared to a control group. QFG helped prevent colitis from progressing to CAC without causing major side effects. This supports the idea that QFG could be used in the future to help prevent CAC.

CD3^+^ T cells serve as indicators of immune function, and the CD4^+^/CD8^+^ ratio is essential for detecting cellular immune dysregulation (Huang et al., 2023). In individuals with CAC, disturbances in CD3^+^ T cell proliferation and CD4^+^/CD8^+^ ratios amplify inflammatory responses and intestinal lesions, thereby facilitating tumor immune evasion (Olguin et al., 2018). Our findings revealed that QFG intervention reduced pro-inflammatory cytokines (IL-1*β*, IL-6, TNF-*α*, and IFN-*γ*), enhanced CD3^+^ T cell propagation, and improved the CD4^+^/CD8^+^ T lymphocyte ratio in mice.

The disruption of the intestinal immune microenvironment plays a crucial role in the transition from IBD to CAC transition, leading to T-cell abnormalities and microbial imbalances (Lu, Shen, Li, & Du, 2024, Wei et al., 2023, Zhang et al., 2023). NOD-like receptors are innate immune pattern recognition receptors located in intestinal epithelial cells, associated with the occurrence of mucosal immune responses. Disruption of the gut microbiota leads to the activation of NOD-like receptor signaling pathways, thereby triggering an inflammatory cycle and exacerbating T cell abnormalities in the inflammatory environment (de Zoete & Flavell, 2013, Jiang et al., 2022). Furthermore, NOD2 activation triggers NF-*κ*B phosphorylation, thereby facilitating the transition from IBD to CAC. These factors reciprocally reinforce a vicious cycle, resulting in tumor immune evasion and ultimately driving colonic dysplasia toward cancer (Wei et al., 2023, Zhang et al., 2023). Current colitis and CAC treatments using prebiotics and probiotics face efficacy challenges due to viability loss and low bioavailability (Zhang et al., 2023). Traditional microbial drugs often target a limited number of bacteria and require multiple prebiotics for effectiveness. TCM enhances immune functions, particularly intestinal immunity (Feng et al., 2022, Wei et al., 2023, Yang et al., 2023). Our study indicated that QFG administration reduces intestinal microbiota disturbances in AOM/DSS-induced CAC mice, aligning their microbiota structure more closely with the control group. Long-term QFG intake did not significantly change mouse intestinal microbiota under non-model conditions. QFG effectively regulates intestinal microbiota, promoting the restoration of normal microbial patterns while exhibiting strong anti-inflammatory effects.

At the phylum level, the F/B ratio was significantly increased in CAC, serving as a potential marker of the severity of gut microbiota alterations. QFG effectively alleviated these CAC-induced intestinal microbiota disturbances, consistent with *α*/*β* diversity observations. This suggests that QFG has a potential role in reducing the relative abundance of gram-positive bacteria, such as Firmicutes, compared to gram-negative such as *Bacteroidetes*, in the context of CAC. Furthermore, microbial variation was assessed at multiple taxonomic levels (class, order, family, and genus) across groups. The AOM/DSS group demonstrated significant changes in 16 bacterial populations; QFG intervention improved 13 out of 16 impacted bacterial strains. Notably, 77% were gram-positive.

QFG, a TCM preparation, exerts multiple target effects. We investigated QFG's primary signaling pathway in CAC inhibition using RNA-seq on AOM/DSS-induced CAC mouse colon tissues following QFG treatment. Results revealed a significant impact on the NF-*κ*B pathway, suggesting QFG suppresses CAC by inhibiting NF-*κ*B p65 activation. NF-*κ*B is a key link between inflammation and tumorigenesis (Wei et al., 2023) by encoding pro-inflammatory cytokines such as TNF-*α*, IL-1*β*, and IL-6 (Cuellar-Nunez et al., 2021, Medicherla et al., 2015), which promote DNA damage and transform colon cells into cancer cells (Zhang et al., 2023). Furthermore, NF-*κ*B p65 phosphorylation enhances these cytokines, which alter T lymphocyte subsets and facilitate the colonization of harmful bacteria, thereby promoting CAC (Medicherla et al., 2015). This is consistent with our RNA-seq data indicating enrichment of the “T cell receptor signaling pathway.” Western blotting and IF confirmed that QFG inhibits NF-*κ*B p65 phosphorylation and nuclear entry, highlighting its therapeutic effect in blocking CAC by targeting colonic NF-*κ*B signaling.

**Fig. 7:**
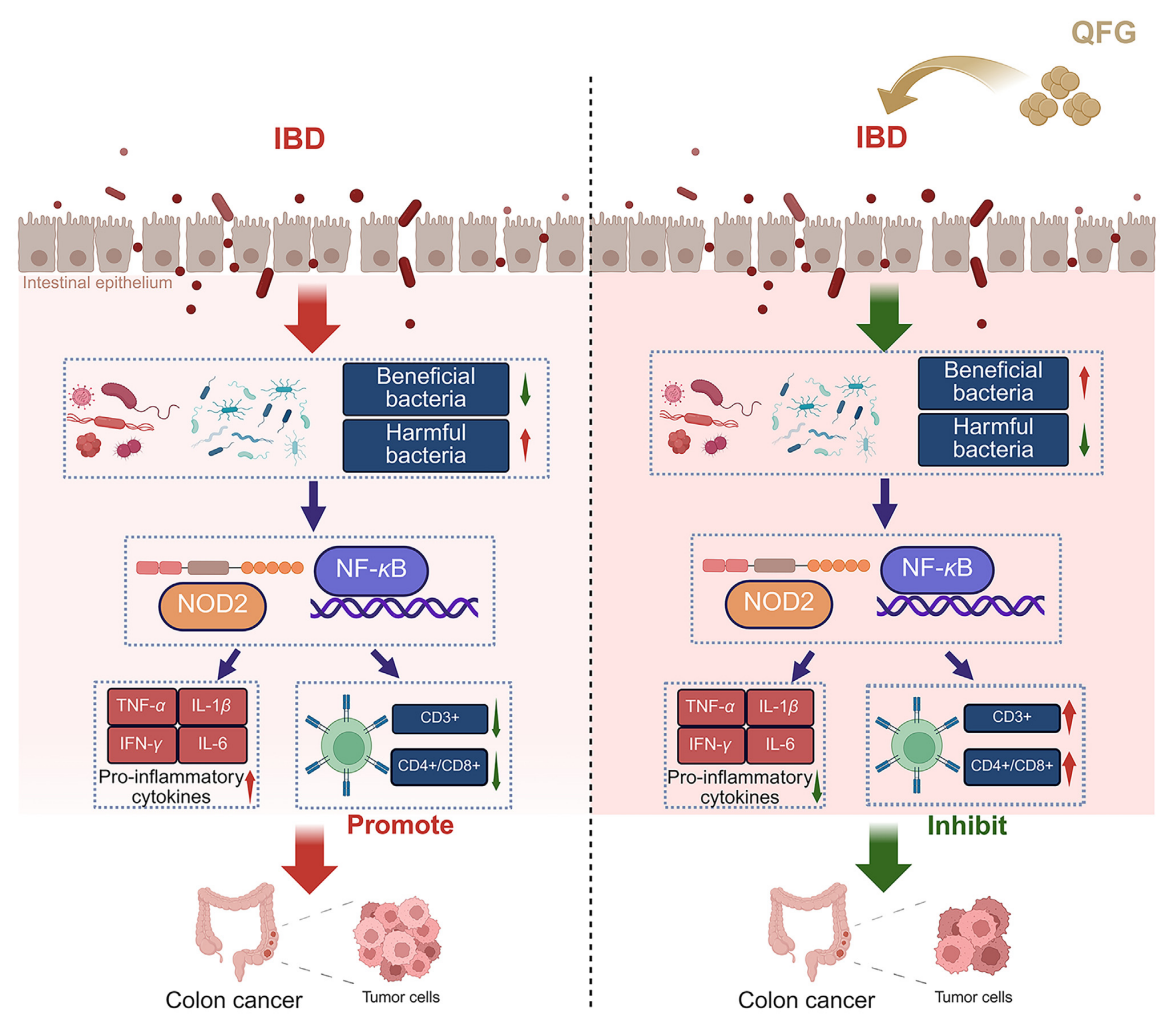
Mechanisms of QFG alleviate immune and microbiota disorder while impacting NOD2/NF-*κ*B signaling pathway to prevent colitis-associated colorectal cancer formation.

Considering the efficacy of QFG on gut microbiota regulation and the complex link between NF-*κ*B activation and the PRRs of the intestinal immune system, we investigated two primary PRR pathways: TLRs and NOD-like receptor signaling routes. RNA-seq results revealed that QFG significantly inhibited NOD-like receptor signaling in CAC tissues. NOD1 detects fragments in gram-negative bacteria, while NOD2 responds to muramyl dipeptide in all bacteria (de Zoete & Flavell, 2013). QFG treatment resulted in a notable reduction in NOD2 expression in CAC mice colonic tissues, while its effect on NOD1 expression was non-significant. These results highlight the importance of the NOD2/NF-*κ*B pathway in QFG's regulation of intestinal microbiota and CAC suppression (Fig. 7).

The correlation analysis between the biological indicators of CAC development and different levels of intestinal microbiota revealed that 13 bacteria exhibited either positive or negative correlations with different characteristics. The abundance of eight bacteria increased due to CAC formation; however, it was inhibited by QFG treatment. These bacteria were positively correlated with markers of disease progression from AOM/DSS-induced IBD to CAC and genes in the NOD2/NF-*κ*B pathway, including Bacilli, Bacillales, Staphylococcaceae, *Staphylococcus*, Lactobacillales, *Aerococcus*, *Alloprevotella*, and *Akkermansia*.

Bacilli (Zhu et al., 2024) and Bacillales harbor many harmful bacteria involved in colitis and tumorigenesis, such as *Listeria monocytogenes* (Asaeda et al., 2021). *Staphylococcaceae* was positively correlated with CRC occurrence (Borges-Canha et al., 2015). *Staphylococcus* is a significant pathogen associated with colitis and CRC (Han & Ding, 2022). Lactobacillales are associated with an increased risk of lung cancer and reduced survival among patients with biliary tract cancer (Zhu et al., 2024), suggesting a potential oncogenic link. *Aerococcus* is significantly enriched in the gut microbiota of patients with late-stage CRC (Alhhazmi et al., 2023). *Alloprevotella* (Li et al., 2024) is a harmful bacterium that contributes to CRC development. Multiple species within *Akkermansia* regulate immune responses and inhibit cancer (Zhang et al., 2023). However, our study revealed a significant increase in *Akkermansia* in the gut microbiota of CAC mice, aligning with robust research outcomes linking its proliferation to CRC (Barot et al., 2024, Crossland et al., 2023). This highlights that a single bacterium can have diverse effects across diseases, and its impact may vary during different stages of the same disease, as exemplified by *Akkermansia*, which may be critical in advancing colorectal inflammation toward cancer in CAC.

Five bacteria exhibited negative correlations with CAC disease markers and NOD2/NF-*κ*B pathway genes, including Clostridiales, Lachnospiraceae, Lachnospiraceae_NK4A136_group, Ruminococcaceae*,* and Muribaculaceae. Following fecal transplantation treatment, Clostridiales significantly decreased in patients with ulcerative colitis (Rees et al., 2022). The Lachnospiraceae (Zhao et al., 2024, Zhao et al., 2023) family and Lachnospiraceae_NK4A136_group (Jia et al., 2024, Yang et al., 2024) are beneficial in colitis and CRC treatment. The Ruminococcaceae family used as a probiotic for CRC treatment revealed an increased abundance of post-effective pharmacotherapy (Lee et al., 2023, Leung et al., 2023). In an experiment involving multiple drug treatments for colitis, the abundance of the Muribaculaceae family significantly increased in the treatment group (Chen et al., 2024, Song et al., 2024).

## 5 Conclusion

In summary, AOM/DSS-induced CAC is associated with recurrent IBD, disrupted immune responses, gut microbiota imbalances, NOD2/NF-*κ*B pathway activation, and pro-inflammatory cytokine secretion. QFG treatment reverses bacterial imbalances, inhibits the NOD2/NF-*κ*B pathway, blocks inflammatory cytokine release, and modulates T cell subsets, thereby preventing CAC development. The safety profile of QFG underscores its potential as a preventive therapy for CAC. Our findings established a network involving IBD, CAC, immune responses, gut microbiota, the NOD2/NF-κB pathway, and QFG, identifying new bacteria associated with CAC. This suggests QFG could be an effective strategy for CAC prevention and treatment, providing new targets in signaling pathways and microbiota for further exploration in CAC management.

Finally, it is important to acknowledge the limitations of our study, including the lack of a clear causal relationship between the drug, microbiota, and signaling pathways. To address these limitations, future research will need to employ methods such as germ-free mice and transgenic animals to further validate our findings. Additionally, we plan to investigate the key microbiota that play a significant role in the inhibition of CAC by QFG and to uncover new bacteria that have a direct correlation with CAC, thereby offering new insights for the clinical prevention and treatment of CAC.

## CRediT authorship contribution statement

**Bin Huang:** Conceptualization, Methodology, Writing – original draft, Writing – review & editing, Funding acquisition. **Honglin An:** Validation, Formal analysis, Visualization. **Mengxuan Gui:** Investigation, Formal analysis. **Yiman Qiu:** Investigation, Data curation. **Wen Xu:** Formal analysis, Investigation. **Liming Chen:** Investigation. **Qiang Li:** Formal analysis, Investigation. **Shaofeng Yao:** Formal analysis, Investigation. **Shihan Lin:** Formal analysis, Investigation. **Tatyana Aleksandrovna Khrustaleva:** Project administration, Supervision. **Ruiguo Wang:** Conceptualization, Resources. **Jiumao Lin:** Supervision, Resources, Funding acquisition.

## Declaration of competing interest

The authors declare that they have no known competing financial interests or personal relationships that could have appeared to influence the work reported in this paper.
