# Supplementary material for: Qingjie Fuzheng Granule prevents colitis-associated colorectal cancer by inhibiting abnormal activation of NOD2/NF-κB signaling pathway mediated by gut microbiota disorder
Source: Chin Herb Med. 2025 Apr 3;17(3):500–12. doi: 10.1016/j.chmed.2025.04.001 (PMC12301919; doi:10.1016/j.chmed.2025.04.001)
Supplement: Supplementary Data 1 [file mmc1.docx]

**Supplementary materials**

**
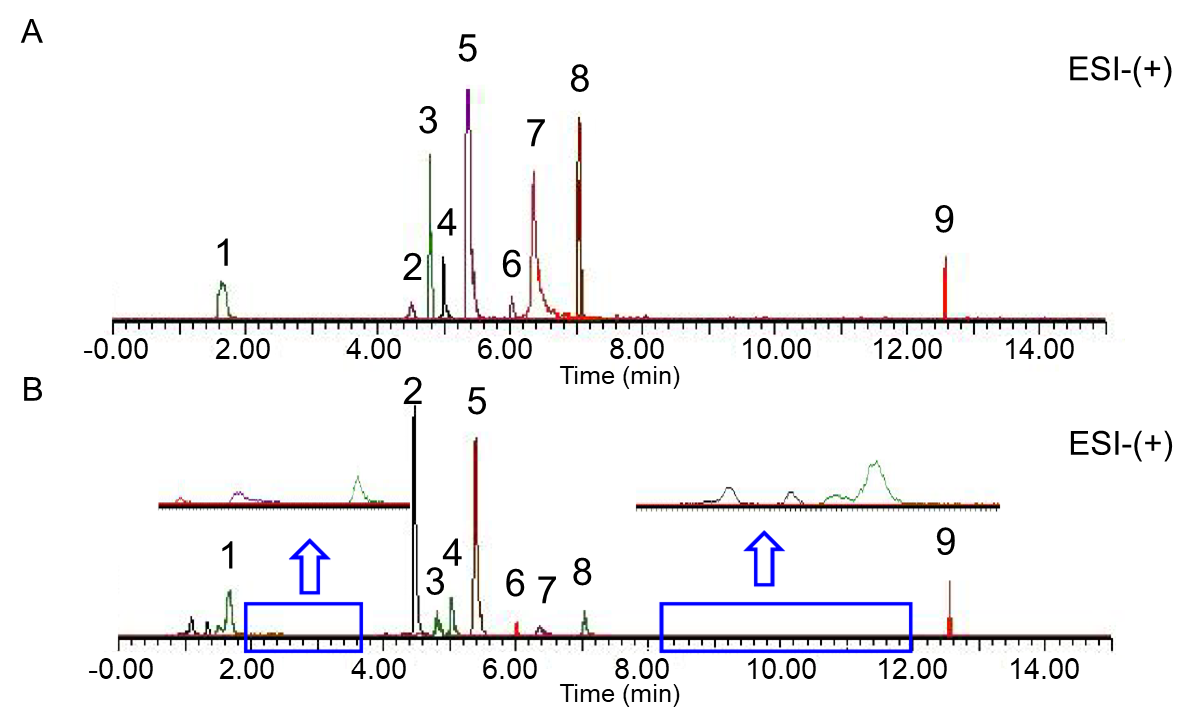
**

**Fig. S1. Total MRM ion chromatogram of reference substances (A) and QFG (B).** Peaks are labeled as follows: 1. Hordenine, 2. Scutellarin, 3. Asperulosidic acid, 4. Asperuloside; 5. Ononin, 6. Astragaloside Ⅳ, 7. Baicalein, 8. Astragaloside, 9. Ursolic acid.


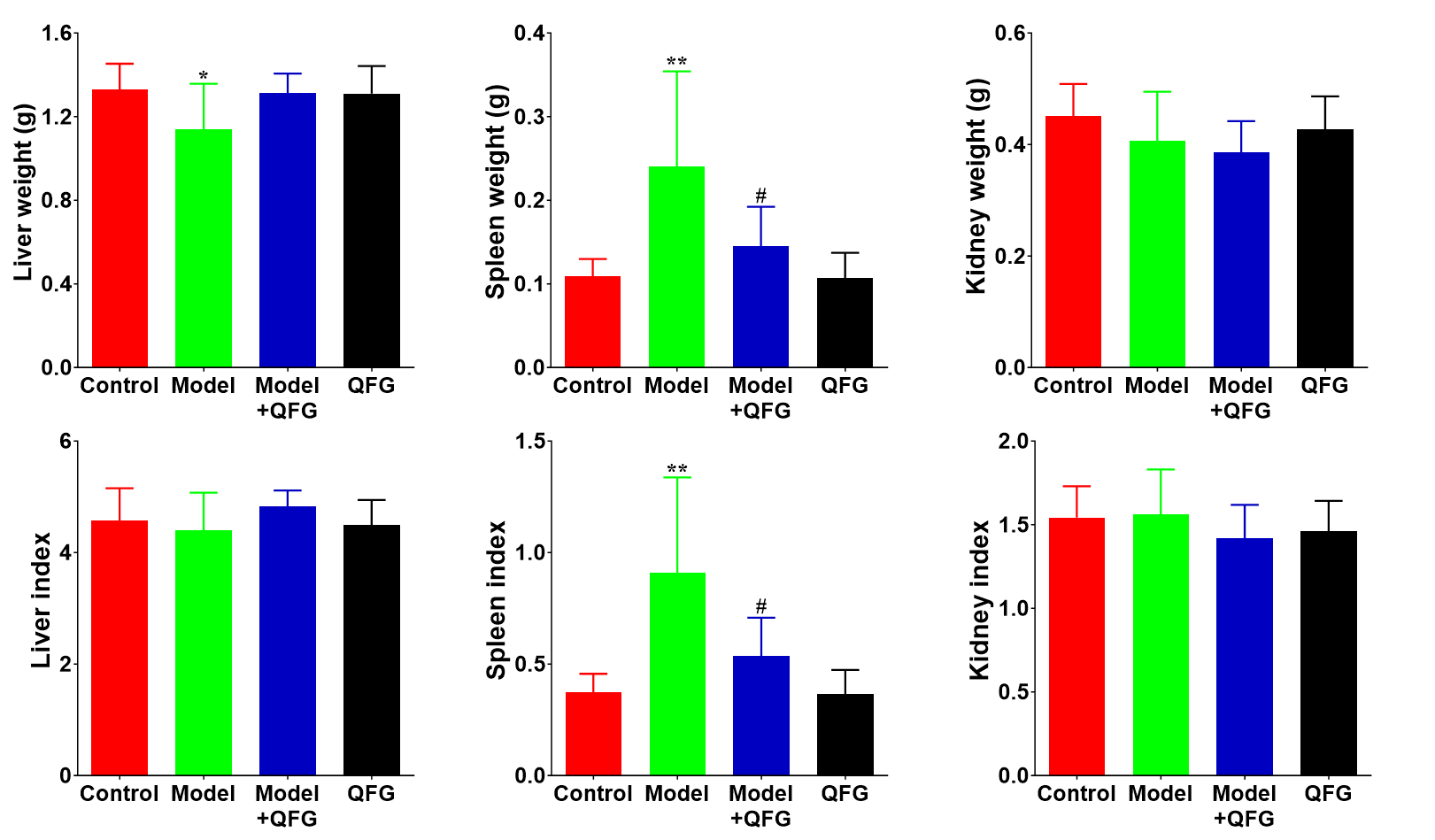


**Fig. S2. Liver, spleen, kidney weight and organ index.** Games–Howell test or Tukey's test and One-way ANOVA were used to determine the statistical significance: ^*^*P* < 0.05, ^**^*P* < 0.01 *vs* control group; ^#^*P* < 0.05 *vs* model group.

**Table S1. Calibration curves of detected compounds and the calculated content of QFG.**

| No. | Analytes | *t_R_* (min) | MS ^1^(*m*/*z*) | MS^2^ (*m*/*z*) | CV (V) | COE (eV) | ES+/- | Linear equation | Content (mg/g) |
| --- | --- | --- | --- | --- | --- | --- | --- | --- | --- |
| 1 | Hordenine | 1.36 | 166.1 | 103.05 | 40 | 22 | ES^+^ | *Y* = 421 314*X* – 14 319 | 0.776 |
| 2 | Scutellarin | 4.49 | 463.07 | 287.1 | 40 | 22 | ES^+^ | *Y* = 114 641*X* – 40 052 | 1.552 |
| 3 | Asperulosidic acid | 4.77 | 455.18 | 275.02 | 40 | 18 | ES^+^ | *Y* = 45 303*X* – 1 733 | 0.248 |
| 4 | Asperuloside | 5.02 | 437.00 | 303.01 | 40 | 20 | ES^+^ | *Y* = 57 474*X* + 8 106 | 0.517 |
| 5 | Ononin | 5.02 | 431.1 | 269.02 | 40 | 20 | ES^+^ | *Y* = 146 814*X* – 12 129 | 2.484 |
| 6 | Astragaloside IV | 6.03 | 807.4 | 627.3 | 40 | 48 | ES^+^ | *Y* = 50 407*X* – 4 044 | 0.518 |
| 7 | Baicalein | 6.35 | 271.05 | 123.01 | 40 | 30 | ES^+^ | *Y* = 88 071*X* + 3 218 | 0.103 |
| 8 | Astragaloside Ⅰ | 7.04 | 849.4 | 669.4 | 40 | 48 | ES^+^ | *Y* = 68 004*X* + 9 646 | 0.082 |
| 9 | Ursolic acid | 12.2 | 455.19 | 406.98 | 40 | 20 | ES^-^ | *Y =* 66 567*X +* 4 591 | 0.062 |
